# Phase Deflectometry for Defect Detection of High Reflection Objects

**DOI:** 10.3390/s23031607

**Published:** 2023-02-01

**Authors:** Xian-Ming Cheng, Ting-Ting Wang, Wen-Bin Zhu, Bai-Di Shi, Wei Chen

**Affiliations:** College of Mechanical and Electrical Engineering, Hohai University, Changzhou 213022, China

**Keywords:** phase deflection, surface gradients, double surface

## Abstract

A method for detecting the surface defects of high reflection objects using phase deflection is proposed. The abrupt change in the surface gradient at the defect leads to the change in the fringe phase. Therefore, Gray code combined with a four-step phase-shift method was employed to obtain the surface gradients to characterize the defects. Then, through the double surface illumination model, the relationship between illumination intensity and phase was established. The causes of periodic error interference were analyzed, and the method of adjusting the fringe width to eliminate it was proposed. Finally, experimental results showed the effectiveness of the proposed method.

## 1. Introduction

There are a large number of highly reflective objects in industrial products, such as mobile phone glass covers, polished metal, rearview mirrors, curved glass, etc. Product quality testing is an essential step before these items leave the factory. Machine vision is widely used in the defect detection of industrial products because of its advantages of low cost and high precision [1]. However, since it is difficult to distinguish non-destructive defects from destructive defects, the effect of traditional machine vision detection methods in detecting high reflection objects is not ideal. Some scholars suggested using photometric stereo vision or the three-dimensional point cloud measurement method to detect destructive defects [2,3,4]. However, photometric stereo vision is suitable for the surface defect detection of diffuse objects. On the other hand, due to the large amount of data and long detection time, the three-dimensional point cloud measurement method is not suitable for rapid detection. In addition, ultrasonic imaging has also been applied to the field of nondestructive testing. Weng et al. [5] analyzed acoustic scattering to identify complex objects in real time, and this study had the potential to be applied to the field of defect detection. Walker et al. [6] showed that the quality of ultrasonic imaging depends on the wavelength of the acoustic wave, which lays the foundation for ultrasonic detection for nondestructive testing. In this paper, phase deflectometry (PD) was used to detect surface defects on highly reflective objects, which is suitable for the high-speed automated inspection of high-volume products. Moreover, PD does not require calibration of the equipment for sound velocity as in ultrasound imaging, and therefore has better detection stability.

PD uses the principle of specular reflection to calculate the surface gradient of the measured object from the phase information of the projected fringes [7]. The fringe modulation is sensitive to the height information, but insensitive to non-destructive defects such as dirt. The destructive defects can be detected based on gradient mutations. However, when the measured object is a transparent object with two surfaces, incoherent superpositions are formed between the upper and lower surfaces, which affect the extraction of the principal phase value. In addition, non-coherent superposition can cause the reflected light brightness to exceed the response interval of the camera.

Among the numerous works that have investigated the problem of incoherent superposition, four points of view were distinguished [8,9]. The first one consists of using an ultraviolet light source to irradiate the measured object to suppress the reflection interference from a lower surface. Ultraviolet light is used to calculate the upper surface phase of the lens without the interference of back surface reflection and then accurately reconstruct the three-dimensional shape of the lens [10]. However, in this method, since there is no suitable spatial light modulator available in the required wavelength range, a moving line is used instead of a phase-shifted sinusoid. In addition, the extra ultraviolet camera increases the hardware cost of the system.

Another way to deal with the problem is to eliminate the lower surface reflection using Gray code and line shift. A fixed threshold is adopted to remove the bottom reflection according to the different reflected light intensity of the upper and lower surfaces [11]. However, when the display resolution is 1024 × 768 pixels, the method needs to generate 40 encoded images, including 22 vertical images and 18 horizontal images, which greatly increases the number of projection images and the detection time.

In the third approach, efforts are made to avoid the overlap of fringe signals on upper and lower surfaces. A narrow line shift scanning deflectometry is proposed to obtain the reflection fringes on the separated surfaces [12], realizing 20 μm roughness measurement of grinding surface. This method avoids the overlap of fringe signals and has high measurement accuracy. Due to linear scanning, some areas cannot be detected and the measurement time is long.

The fourth is to separate the phase from the front and rear surfaces [13]. Without special equipment, high precision detection is obtained through the combination of a spectral estimation algorithm and projected multi-frequency fringes [14]. However, the fringe image projection of up to 2000 frames not only increases the projection time, but also requires Fourier transformation on all pixels point by point, which greatly increases the detection time. Combining the traditional phase-shifting technology with known fringes can effectively eliminate the influence of parasitic reflection [15,16].

On the other hand, when the intensity input to the camera exceeds the maximum value, the camera image may saturate, leading to phase diagram errors. Researchers have tried various methods to avoid the image saturation, such as adjusting the projection intensity [17] and decreasing the exposure time. Typically, adjusting the projection intensity requires acquiring a large number of images, which is time consuming. However, decreasing the exposure time may contribute to the reduction of the distortion streak modulation system, which may reduce the detection accuracy. Notably, several reports revealed that the phase error problems because of the saturation of images can be solved even for the saturated images [18]. Hu et al. [19] analyzed the phase error due to the saturation of partial stripe intensity and defined a saturation factor K for describing the image saturation [20]. Chen et al. [21] found that for the same phase-shifted stripe patterns, if the stripe period P is even, the N = P × *n*/2 (*n* = 1, 2, …) steps algorithm can recover the phase accurately even if the stripe patterns are saturated. Qi et al. [22] performed a comprehensive analysis of the phase errors caused by the image saturation, and they concluded that when the integer period sampling (IPS) condition is met, no phase error is introduced.

In this paper, a phase deflection method for surface defect detection of high reflection objects is proposed. For the non-coherent superposition of upper and lower surfaces, this paper proposed a method to eliminate the interference by adjusting the projected stripe width. Compared with previous research works, the proposed method can quickly detect the damaging defects on the surface of two-surface objects without increasing the hardware cost. This paper is organized as follows: Section 2 introduces the basic theory of detection, including the demodulation phase and phase unwrapping. Section 3 analyzes the error sources and the elimination method of detecting double surface objects. In Section 4, the effectiveness of the method was proved by the experimental results. Finally, conclusions are given in the last section.

## 2. Theoretical Basis of PD and Surface Defect Detection

### 2.1. Principle of PD for Defect Detection

**Figure 1:**
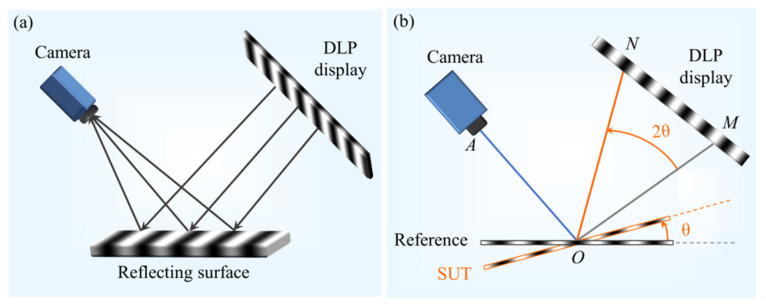
(**a**) Schematic diagram of PD system; (**b**) basic principle of PD for defect detection.

The PD system consists of a display, a reflective object (surface of the object to be tested) and a camera, as shown in Figure 1a. The structured light projected from the display illuminates the surface under test (SUT). After the test surface was modulated, the light containing three-dimensional information was captured by the camera.

The gradient information of the reflecting surface can be obtained by extracting and dephasing the phase of the mirror image. The gradient at the defect changes sharply, and the defect can be determined according to this principle.

The geometric principle of PD for defect detection systems is shown in Figure 1b. In the PD system, when a certain point *M* on the display is lit, the light is reflected into the camera *A* along *MO* through the surface of the measured object. If the SUT is defective (equivalent to the rotation of the reflecting surface), the light received by the camera is the deflected spot *N* after the deflection. For the non-destructive defects such as slight surface dirt, the light reflection angle does not change, i.e., it does not affect the gradient detection of the measured object surface. According to this principle, PD for defect detection system can be used to detect damaged defects on the surface of the tested object without being disturbed by dirt.

### 2.2. Demodulation of Sinusoidal Fringe

In the phase-shifting method, the folded phase principal value is obtained using graphical calculation of multiple sinusoidal fringes with different phase-shifts. The equal phase stepping method is usually adopted, i.e., the fringe light moves *N* times in one direction, and the phase-shifted fringe pattern *I_n_*(*x*,*y*) is a one-dimensional sinusoidal waveform [23]:

(1) $$I_{n}(x,y)=a(x,y)+b(x,y)\cos\left[\varphi (x,y)+\frac{2\pi (n-1)}{N}\right],$$

where *a*(*x*,*y*), *b*(*x*,*y*) and *φ*(*x*,*y*) are the background, modulation and phase, respectively.

In order to obtain continuous absolute phase values, it is necessary to determine the periodic sequence at the phase truncation, which is usually represented by *k*. By adding the periodic sequence 2*k*(*x*,*y*)*π*, the unwrapped phase values are obtained on the basis of folded phase *φ*(*x*,*y*). The expression of one-dimensional phase unwrapping can be expressed as:

(2) Φ(x,y)=φ(x,y)+2k(x,y)π,

where *Φ*(*x*,*y*), *φ*(*x*,*y*) and *k*(*x*,*y*) represent absolute phase, wrapped phase and periodic series, respectively.

Equation (2) was used for one-dimensional phase unwrapping. The two-dimensional phase expansion method can be further derived from the one-dimensional phase unwrapping principle. The unwrapping process is as follows:

(1) Select the phase unwrapping origin, and start from the origin to perform a one-dimensional column phase unwrapping to obtain the phase unwrapping value of the column where the point is located.

(2) Take the phase value of each point in the column as the initial value, and then perform phase unwrapping on each row to obtain the relative two-dimensional phase unwrapping.

(3) Correct the deviation of the unwrap phase, i.e., add a fixed value to all phases to obtain the true value of the 2D phase expansion.

**Figure 2:**
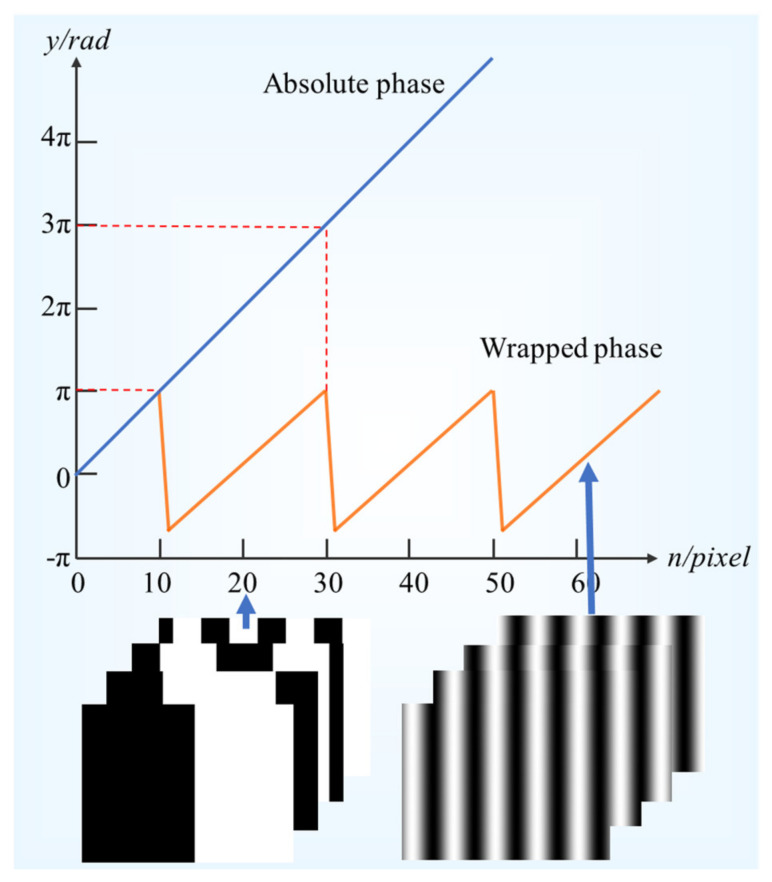
Decoding diagram of Gray code combined with phase−shift code.

According to Equation (2), the absolute phase in the x- or y-direction is determined by the wrapped phase and the number of period steps in which the wrapped phase is located. The wrapped phase can be derived from Equation (1), while the number of period steps is determined by encoding the image with Gray code. Using Gray code to unwrap the phase does not cause error accumulation. Moreover, combined with phase-shift code, the coding bits of Gray code are reduced and the decoding speed is accelerated. Gray code is similar to binary coding in that each pixel of the image is binary coded by black and white stripes, thus determining the number of period steps where the wrapped phase is located, i.e., *k*(*x*,*y*) in Equation (2). Therefore, the unwrapping process of the one-dimensional phase is shown in Figure 2.

According to the relationship between phase and gradient, the gradient distribution in *x* direction and *y* direction on the SUT can be obtained as:

(3) $$g_{x}=\tan\theta_{x}=\frac{P_{x}\Delta \Phi_{x}}{4\pi |OM|},$$

(4) $$g_{y}=\tan\theta_{y}=\frac{P_{y}\Delta \Phi_{y}}{4\pi |OM|},$$

where g*_x_*, *θ_x_*, Δ*θ_x_* and *P_x_* represent the gradient, the deflection angle of the measured surface, the phase difference and the fringe period in the *x* direction, respectively. Similarly, g*_y_*, *θ_y_*, Δθ*_y_* and *P_y_* represent the corresponding parameters in the *y* direction. The absolute phase is positively correlated with the surface gradient of the measured object. The gradient at the defect changes suddenly, thus the gradient can be used to characterize the defect.

## 3. Error Analysis and Elimination

### 3.1. Model of Double Surface Reflection

When there is back reflection, in addition to receiving the light reflected from a point on the upper surface, the camera also receives the light reflected from a point on the lower surface according to the reversible nature of the optical path. The light intensity signal received by the CCD camera can be expressed as [24]:

(5) $$I_{accept}=I_{front}\left(n\right)+I_{rear}\left(n\right),$$

where *I*_accept_ is the total light intensity received by the CCD, *I*_front_ and *I*_rear_ are the reflected light intensities of the front and rear surfaces and *n* is the number of the current phase-shift steps. Therefore, when the thickness of the measured object is uniform, the brightness of each point is superimposed except the point on the edge.

**Figure 3:**
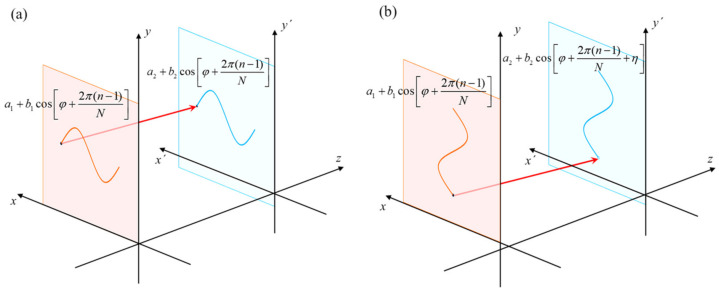
Incoherent superposition of upper and lower surfaces. (**a**) Superposition of sinusoidal fringes in *x* direction; (**b**) superposition of sinusoidal fringes in *y* direction.

In order to solve the global gradient, the fringe images in *x* and *y* directions need to be projected sequentially. When the fringe image in the *x* direction is projected, the phase of the sinusoidal fringe pattern on the upper surface and the lower surface is consistent, as shown in Figure 3a. When projected in the *y* direction, the phase is different, as shown in Figure 3b.

If *a*_1_(*x*, *y*) and *a*_2_(*x*, *y*) represent the background light intensity of the front and rear surface, respectively, *b*_1_(*x*, *y*) and *b*_2_(*x*, *y*) represent their modulation degree, *φ*(*x*, *y*) represents the phase modulated by the surface of the measured object [25,26] and *N* represents the total number of phase-shift steps, then

(6) $$I_{}{}_{\mathrm{front}}(x,y)=a_{1}(x,y)+b_{1}(x,y)\cos\left[\varphi (x,y)+\frac{2\pi (n-1)}{N}\right],$$

(7) $$I_{}{}_{\mathrm{rear}}(x,y)=a_{2}(x,y)+b_{2}(x,y)\cos\left[\varphi (x,y)+\frac{2\pi (n-1)}{N}+\eta \right],$$

where *η* indicates the phase-shift due to the height difference between the front and rear surfaces.

If the *x* direction fringe image is projected, *η* is 0. In Equations (6) and (7), *a*_1_(*x*,*y*) and *a*_2_(*x*,*y*) denote the background light intensity, which can be represented by *a*_12_. If we write *b*1 (*x*,*y*) and *b*_2_ (*x*,*y*) as *b*_1_ and *b*_2_, and *φ*(*x*,*y*) as *φ*, then Equation (5) is expressed as:

(8) $$I_{\mathrm{accept}}=a_{12}+b_{1}\cos\left[\varphi +\frac{2\pi (n-1)}{N}\right]+b_{2}\cos\left[\varphi +\frac{2\pi (n-1)}{N}+\eta \right],$$

If *η* = *π*, the modulation of the upper and lower surfaces differs by half a cycle, and

(9) $$I_{\mathrm{accept}}=a_{12}+(b_{1}-b_{2})\cos\left[\varphi +\frac{2\pi (n-1)}{N}\right].$$

In this case, the upper and lower surface regulating system cannot be equal, otherwise the received stripes will lose their sinusoidality.

When *η* ≠ *π*:

(10) $$I_{\mathrm{accept}}=a_{12}+b_{1}{}_{2}\cos\left[\varphi +\frac{2\pi (n-1)}{N}+\delta \right],$$

where *δ* indicates the phase-shift due to incoherent superposition between the upper and lower surfaces.

### 3.2. Phase Error Elimination

As can be seen from Equation (10), the superimposed stripes are still sinusoidal, only the amplitude and phase have changed, with no change in period. If the width of the projected stripe is equal to the phase-shift caused by the thickness difference between the upper and lower surfaces, i.e., *η* = 2*π* (Equation (8)), the superimposed stripe changes only in amplitude, and Equation (10) can be formulated as:

(11) $$I_{\mathrm{accept}}=a_{12}+b_{1}{}_{2}\cos\left[\varphi +\frac{2\pi (n-1)}{N}\right],$$

where *a*_12_ and *b*_12_ determine the value range of *I*_accept_. *A*_12_ is the DC fundamental component of sinusoidal fringe, and *b*_12_ represents the amplitudes of upper and lower surfaces, respectively. Therefore, the upper boundary *I*_max_ and lower boundary *I*_min_ are as follows:

(12) $$\left\{\begin{array}{l} I_{\max}=a_{12}+b_{12}\\ I_{\min}=a_{12}-b_{12} \end{array}\right.,$$

**Figure 4:**
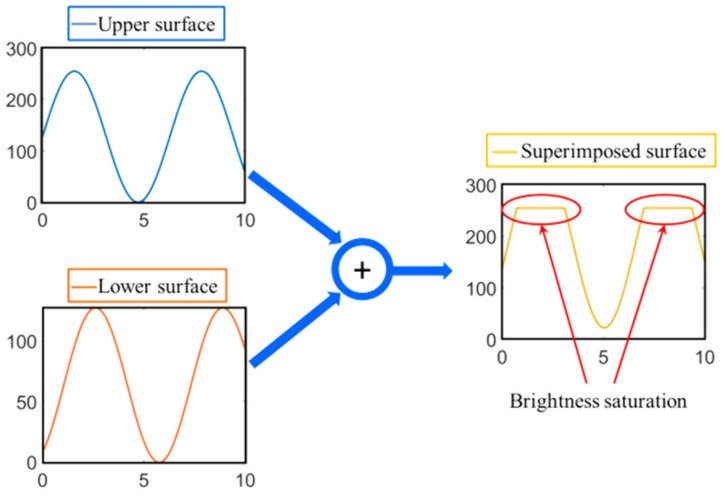
Sinusoidal fringes are superimposed to form the top platform and phase mutation occurs.

Usually, the sinusoidal fringes on the upper surface just fill the response range of the camera to ensure detection accuracy. However, when the DC fundamental component moves up *a*_2_ units, the brightness values beyond the camera response range are truncated at 255, as shown in Figure 4.

From the conclusions drawn by Qi et al. [22], it is known that when the IPS condition is satisfied, no image saturation error is introduced. Therefore, the phase error can be avoided when the projected stripe width is adjusted to the thickness of the upper and lower surfaces of the measured object and the IPS condition is satisfied.

## 4. Experiment and Discussion

**Figure 5:**
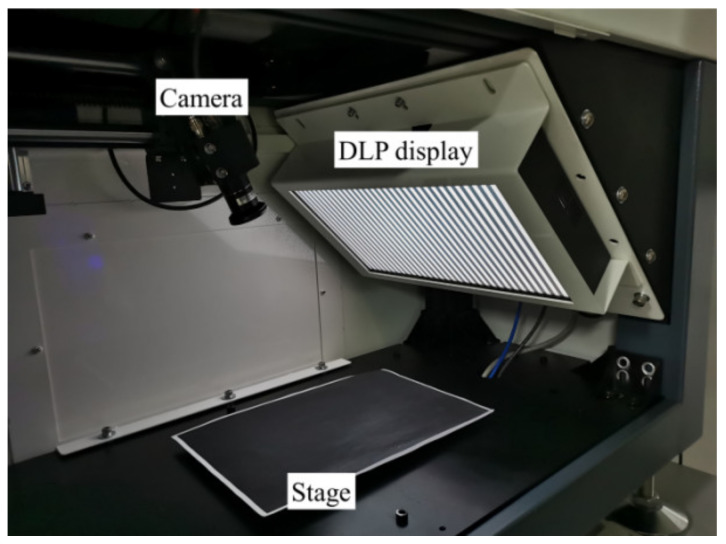
PD defect detection system.

In order to verify the feasibility of using PD to detect surface damaging defects of highly reflective objects, we apply it to the actual PD system. The test system in Figure 5 is composed of a digital light processor (DLP) screen (P-DIS-T15-S, produced by Rsee, Dongguan, China, resolution 1920 × 1080, pixel pitch 0.13 mm), stage and CCD camera (MV-CA050- 20GM, manufactured by Hikvision in Shenzhen, China, resolution 1280 × 960, image element size 3.75 × 3.75 μm) et al. The display area of the screen is 256 × 144 mm. The tested objects include polished metal plate, automobile door bowl, mobile phone glass cover plate and rear-view mirror, which are plane objects, concave objects, plane objects with double surfaces and convex objects with double surfaces. These examples provide case studies for the application of the methods presented in this paper.

### 4.1. Defect Detection Experiment of Polished Metal Plate

**Figure 6:**
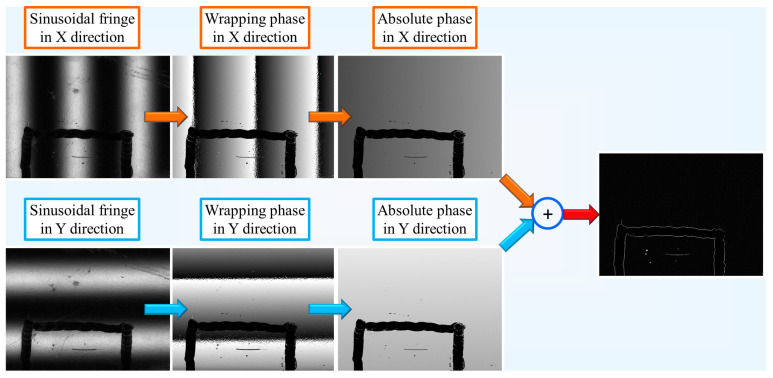
Transformation process of obtaining defect result.

During the test, we placed the polished metal plate on the stage and set the DLP to white screen display. Then, we adjusted the aperture of the camera to make the imaging almost overexposed (which can suppress the dirt). Finally, the fringe pattern was projected sequentially. There were eight phase-shifting fringes, four vertical and four in a horizontal direction. Twelve Gray code images were used to determine the periodic series of sinusoidal fringes to the unwrapped phase, and six in *x* and *y* directions, respectively. The minimum Gray difference was set to 40, which was used to determine the binary image. The experimental results showed that the image quality was higher when the sinusoidal fringe width was 128 pixels and the camera exposure time was 25,000 μs. First, the wrapped phases in both directions were obtained from the sinusoidal stripe maps in the *x*- and *y*-directions, respectively. Then, the number of period steps of the folded phase was determined using Gray’s code. The Gray code was similar to the binary encoded image, where each pixel region of the image was encoded by black and white stripes, and thus the number of period steps in Equation (2) was determined. Further, the gradients in the *x*- and *y*-directions were calculated and the global gradients were synthesized according to Equations (3) and (4). Finally, the gradient map was grayscale transformed to obtain the final defect map. The unwrapping process is shown in Figure 6.

**Figure 7:**
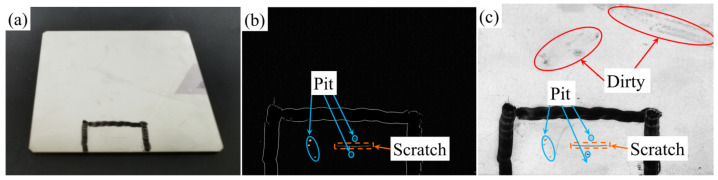
Polished metal plate (**a**), test results (**b**) and bright field image (**c**).

Figure 7a–c show the real object, detection results and bright field images, respectively. It can be clearly seen from Figure 7c that there are dirt, pits and scratches on the surface of the tested object, which are difficult to distinguish. Figure 7b effectively detects damaging defects without being disturbed by dirt. The experimental results show that the proposed method is suitable for the detection of surface damage defects of plane high reflection objects.

### 4.2. Experiment on Defect Detection of Automobile Door Bowl

**Figure 8:**
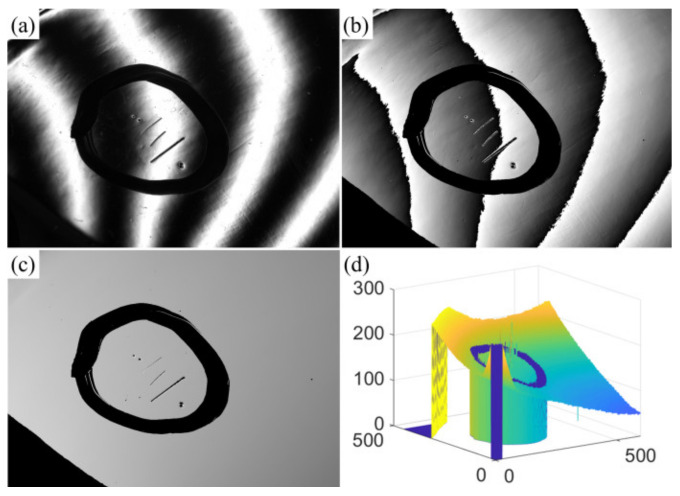
The process of obtaining absolute phase diagram in x direction. (**a**) Sinusoidal fringe, (**b**) wrapped phase, (**c**) absolute phase and its 3D view (**d**).

The experiment of the door bowl was carried out to verify the applicability of the proposed method to the detection of concave objects. Similarly, when the width of sinusoidal fringe was set to 128 pixels, the detection effect was the best. The folded phase shown in Figure 8b was solved using the *x* direction phase-shift code shown in Figure 8a. The absolute phase is shown in Figure 8c. Figure 8d is a three-dimensional view of the global phase. It can be seen from the figure that the unfolding phase is flat and there is no sudden change, which can be used for defect detection.

**Figure 9:**
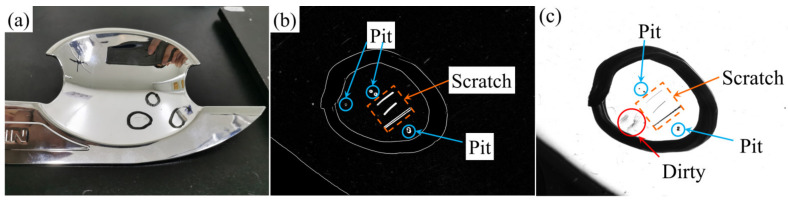
Automobile door bowl (**a**), test results (**b**) and bright field image (**c**).

Figure 9a shows the real object of the door bowl. Comparing the phase deflection defect detection results in Figure 9b with the bright field image in Figure 9c, it can be seen that the proposed method can not only filter the dirt interference, but also detect the pit defects not detected in the bright field image, and the detection accuracy is higher.

### 4.3. Experiment of Rearview Mirror Defect Detection

**Figure 10:**
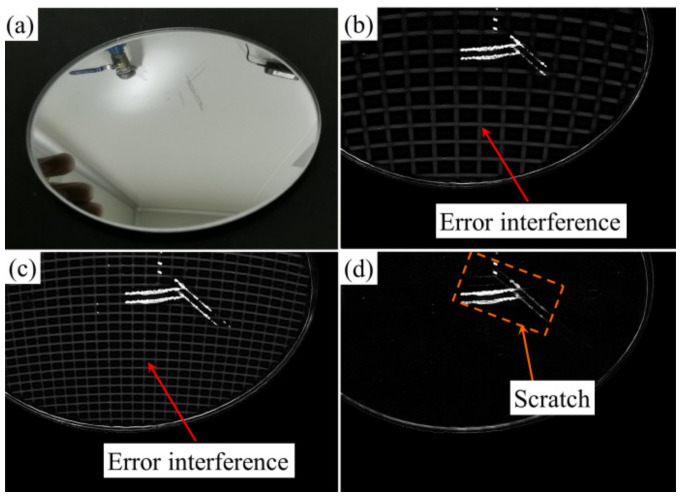
Rearview mirror (**a**) and results of different stripe widths. (**b**) 256 pixels, (**c**) 128 pixels and (**d**) 32 pixels.

To illustrate that the proposed method is applicable to convex objects with double surfaces, as shown in Figure 10a, the rearview mirror was used in this experiment. Figure 10b–d show the results when the projection fringe width was 256, 128 and 32 pixels, respectively. As can be seen from Figure 10b,c, it cannot be used for detection due to periodic error interference. When the width of the projected fringe was equal to 32 pixels of the phase-shift difference between the upper and lower surfaces, there was basically no error interference, and the scratch was also detected. Experiments show that PD can be used for mirror surface defect detection.

### 4.4. Defect Detection Experiment of Mobile Phone Glass Cover Plate

**Figure 11:**
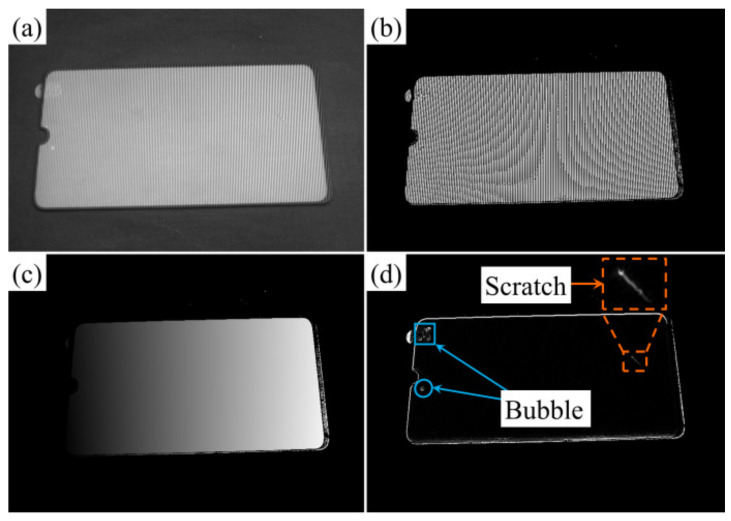
Process of mobile phone glass cover algorithm. (**a**) Sinusoidal fringe, (**b**) wrapped phase, (**c**) absolute phase and (**d**) detection result.

Figure 11a–d are the *x* direction sinusoidal fringe pattern, folded phase pattern, global phase pattern and detection result diagram when the projection fringe period was 16 pixels. It can be seen that when the width of the projection fringe was 16 pixels (just equal to the phase-shift difference between the upper and lower surfaces), there was no error interference in the detection result. In the PD test results, not only scratches but also coated bubbles were detected, because the bubbles reflected light and affected the surface gradient. On the other hand, it illustrated that the PD detection system is not suitable for the detection of surfaces containing reflective dirt.

## 5. Conclusions

In this paper, a PD method for detecting surface damage defects of highly reflective objects is proposed, which is verified by experiments on a polished metal plate, an automobile door bowl, a rearview mirror and a mobile phone glass cover plate. The main idea was to use the gradient graph to represent the defect graph, which was based on the principle that the gradient at the defect changed suddenly. In order to solve the incoherent superposition interference of double-sided objects, a method of adjusting the fringe width to eliminate the error interference is proposed. This method does not require the addition of special equipment. Without damaging the surface of the measured object, the influence of incoherent superposition can be effectively eliminated, and the application scope of polarization mode dispersion can be extended to the surface defect detection of double surface objects and the method proposed in this paper is only applicable to the defect detection of continuous surfaces.

## Data Availability

Not applicable.
